# Factors Correlated with Post-Surgery Residual Carcinoma in Cases of Breast Cancer Incidentally Found via Vacuum-Assisted Excision: An Ultrasound Perspective

**DOI:** 10.3390/diagnostics15192549

**Published:** 2025-10-09

**Authors:** Qiongchao Jiang, Simin Li, Guoxue Tang, Xiaofeng Guan, Wei Qin, Huan Wu, Haohu Wang, Xiaoyun Xiao

**Affiliations:** Department of Ultrasound, Sun Yat-sen Memorial Hospital, Sun Yat-sen University, 107# Yanjiang Xi Road, Guangzhou 510120, China; jiangqch3@mail.sysu.edu.cn (Q.J.); lism53@mail2.sysu.edu.cn (S.L.); tanggx5@mail.sysu.edu.cn (G.T.); guanxf3@mail.sysu.edu.cn (X.G.); qinwei7@mail.sysu.edu.cn (W.Q.); wuhuan@mail.sysu.edu.cn (H.W.); drwang0914@163.com (H.W.)

**Keywords:** breast cancer, ultrasound, breast imaging reporting and data system (BI-RADS), vacuum-assisted excision, breast-conserving surgery

## Abstract

**Objectives**: To identify factors correlated with post-surgery residue in cases of breast cancer incidentally found via vacuum-assisted excision (VAE). **Methods**: A total of 6083 patients were enrolled in a retrospective study. Ultrasound evaluation and ultrasound-guided VAE were performed on these patients. According to the pathology of VAE, 53 patients with incidentally found breast cancer were included in the final analysis. Either breast-conserving surgery or mastectomy was performed. The maximal diameter, depth, location, BIRADS category, and Adler’s grade of all lesions before VAE was reviewed and recorded. VAE and post-surgery pathologies were used as gold standards. Either Pearson’s chi-square test or Fisher’s exact test was used for comparison of categorical variables. **Results**: The mean age of the enrolled patients was 49 years (IQR: 43–55 years). The mean maximal diameter of the lesions was 11.3 mm (IQR: 7–15 mm). There were twenty-eight ductal carcinomas in situ, twelve invasive ductal carcinomas, five lobular carcinomas in situ, two invasive lobular carcinomas, four intraductal papillary carcinomas, and two mucinous carcinomas. Post-surgery pathology showed 15 cases with residual cancer and 38 cases with no residual cancer. The maximal diameter, depth, and pathology derived via VAE were statistically correlated with post-surgery residue (*p* < 0.05). **Conclusions**: Small incidentally found noninvasive carcinomas located comparatively deep in the breast could be totally excised by ultrasound-guided vacuum-assisted excision. Both large and superficially invasive carcinomas were more likely to be associated with residue.

## 1. Introduction

The ultrasound-guided vacuum-assisted procedure is both minimally invasive and time-saving. Most patients choose this procedure for excision of probably benign breast lesions, owing to its cosmetic outcome. Previous studies have shown that complete excision without residual tissue is possible in most cases [1,2,3]. Even benign phyllodes tumors and intraductal papillomas, which typically require invasive surgical excision, can also be removed via ultrasound-guided vacuum-assisted excision (VAE) [4,5,6]. Nevertheless, previous studies have also shown that a complete removal indicated by ultrasound imaging does not necessarily mean a histologically complete removal, and it has been suggested that only minimally invasive excision techniques should be applied in cases of benign breast tumors [7]. In most studies, the use of regular-interval follow-up to confirm complete lesion excision has also been shown to involve certain limitations.

Furthermore, in recent years, there has been increasing interest in using minimally invasive approaches for local therapy of breast cancer [8,9]. The vacuum-assisted mechanism involves a single insertion of the puncture needle with repeated incisions; hence, it reduces the incidence of needle-tract implantation. However, Michaela Björnström et al. reported that complete excision of small invasive breast cancers was not achieved in any case in which ultrasound-guided VAE was utilized, indicating that this technique lacks reliability for this specific application [10].

In cases of lesions categorized as BI-RADS 3 or BI-RADS 4A by ultrasound, incidental carcinoma might be found using VAE. The question of whether such incidentally found breast cancer could be excised completely is an interesting topic worth exploring. So far as we know, only a few studies have discussed unexpected breast cancer confirmed by VAE. These studies have explored the issue from different perspectives. Kong Y. et al. explored the impact of VAE on surgery options and investigated margin status during breast-conserving surgery in cases of breast cancer which were underestimated by ultrasound [11]. Zhou W. et al. investigated occurrence rates of breast-cancer-mimicking benign lesions (BI-RADS 3 or 4A) and explored the factors responsible for late diagnosis of T2-stage ultrasound-underestimated breast cancer [12].

In contrast to these studies, our retrospective study aimed to analyze correlation between breast cancer incidentally found by VAE and post-surgery residual cancer from a ultrasound perspective, and thus support future selection of tailored treatment.

## 2. Materials and Methods

### 2.1. Patients

This retrospective study was approved by the Medical Ethics Committee of Sun Yat-sen Memorial Hospital, Sun Yat-sen University (Approval No. SYSKY-2024-742-01) on 20 August 2024. The requirement for written informed consent was waived. A total of 6083 patients who consecutively underwent ultrasound-guided vacuum-assisted excision or biopsy between January 2020 and July 2025 were initially enrolled in the study. The inclusion criteria were as follows: (a) the lesions were categorized as BI-RADS 3 or BI-RADS 4A according to the American College of Radiology Breast Imaging Reporting and Data System (ACR BI-RADS 2013), with malignancy likelihood < 10%; and (b) the procedure was carried out with the assumption of complete excision being confirmed by ultrasound. The exclusion criteria were as follows: (a) the lesions were categorized as above BI-RADS 4A; (b) the procedure was carried out for the purpose of diagnostic tissue sampling; (c) the patient had malignancy confirmed by the vacuum-assisted procedure but final surgical pathology was not available; and (d) the patient had more than one malignant lesion confirmed by VAE.

According to ACR BI-RADS, routine follow-up was suggested for lesions categorized as BI-RADS 3. In our hospital, planned excisions of BI-RADS 3 lesions were based on the following reasons: palpable mass, previous personal history of breast cancer, patient anxiety, patient unwillingness/inability to attend follow-up, history of breast cancer in a first-degree relative, or a planned pregnancy.

### 2.2. Data Collection

All patients underwent ultrasound examinations prior to the vacuum-assisted procedure. Ultrasound evaluation and real-time guidance were performed by 2 doctors, each with over 10 years’ experience of breast ultrasound. The ultrasound equipment used in the study was as follows: an Arietta 850 (Hitachi-Aloka Medical, Tokyo, Japan) platform with an L22-2 variable frequency transducer, and an ACUSON Oxana 2 (Siemens Medical Solutions, Erlangen, Germany) machine with an L18-6 variable frequency transducer. All 2D characteristics and color Doppler characteristics of the target lesions were recorded. The maximal diameter, depth (the distance between the skin and the anterior border of the lesion), ACR BI-RADS category, Adler’s grade, and location of the lesion (whether it was located in the areola area or not), as well as the adjacent structures (breast tissue or others), were all recorded.

The device used for vacuum-assisted excision was an EnCor^®^ 7G machine (EnCor^®^, SenoRx, Aliso Viejo, CA, USA). All patients took a supine position. After thorough administration of a local anesthetic, the lesions were completely excised by EnCor. Real-time guidance and instant evaluation by ultrasound were undertaken to attempt complete excision. In cases where malignancy was confirmed by pathology, surgery was performed within one month. Sentinel lymph node biopsy was also performed according to clinical protocol. The pathologies of tissues obtained via vacuum-assisted excision and post-surgery excision were noted.

### 2.3. Statistical Analysis

Continuous variables were expressed as medians with interquartile ranges (IQRs). Categorical variables were expressed as numbers with percentages. For analysis, we carried out transformations of continuous data according to clinical practicability. Lesion size was converted to a categorical variable using 10 mm as a cutoff point, while lesion depth was converted using 5 mm as a cutoff point. Patient age was converted to a categorical variable using 40 years as a cutoff point. The pathology of vacuum-assisted excision was binarily classified as carcinoma in situ or invasive carcinoma. SPSS 27.0 for Windows (SPSS, Inc., Chicago, IL, USA) was used for all statistical analyses. Either Pearson’s chi-square test or Fisher’s exact test was used for comparison of categorical variables. All reported univariate *p*-values were two-sided. *p* < 0.05 was considered statistically significant.

## 3. Results

### 3.1. Pathological Characteristics of All Enrolled Patients

**Figure 1:**
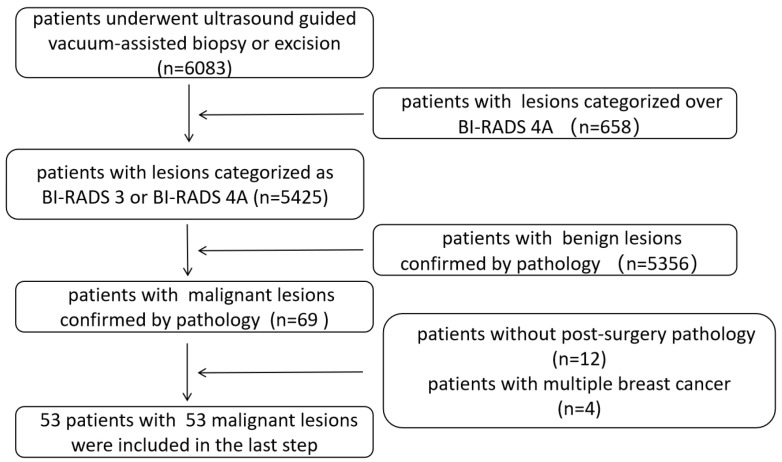
Flow chart of patient enrollment.

Pathological characteristics of 6083 patients who underwent ultrasound-guided vacuum-assisted procedures were reviewed. A total of 658 patients with lesions categorized as BI-RADS 4B or BI-RADS 4C were excluded for biopsy purposes. A total of 69 instances of incidentally found breast cancer were identified. Twelve patients were excluded due to a lack of post-surgery pathology, and four patients with more than one malignant lesion were also excluded. Finally, 53 patients with a total of 53 lesions were enrolled in the retrospective study. The inclusion and exclusion process is shown in Figure 1.

The mean age of the enrolled patients was 49 years (IQR: 43–55 years). Pathological characteristics are summarized in Table 1.

### 3.2. Ultrasound-Pathological Characteristics Correlated with Post-Surgery Residue

Correlations between ultrasound-pathological characteristics of all lesions and post-surgery residue are summarized in Table 2. Thresholds like age, maximal diameter and depth were defined based on epidemiological features of breast cancer in our country and on standard clinical procedures for VAE. The mean maximal diameter of the lesions was 11.3 mm (IQR: 7.0–15.0 mm). The lesions were located at a mean depth of 7.3 mm (IQR: 4.0–10.0 mm). Six lesions were categorized as BI-RADS 3, while forty-seven were categorized as BI-RADS 4A. Only 26 of the 53 patients underwent mammography prior to VAE. Results for these patients were as follows: BI-RADS 1(8), BI-RADS 2(4), BI-RADS 3(4), BI-RADS 4A (6), and BI-RADS 4B (4). Pre-surgery ultrasound was performed on 39 patients; this detected 24 hypo-echoic lesions and 15 mix-echoic lesions in the location of the lesion excised by vacuum-assisted procedure.

All patients underwent breast-conserving surgery. In 38 cases, no residual cancer was found. In 15 cases, the presence of residual cancer was found. Among these cases, there were three invasive carcinomas, two mucinous carcinomas, and ten carcinomas in situ. No lesions were upstaged after comparison of vacuum-assisted-excision and post-surgery pathologies. No lymph node metastasis was found by sentinel node biopsy. All the incidentally found breast cancers were at stage 0(TisN0M0) or stage 1A(T1N0M0).

## 4. Discussion

**Figure 2:**
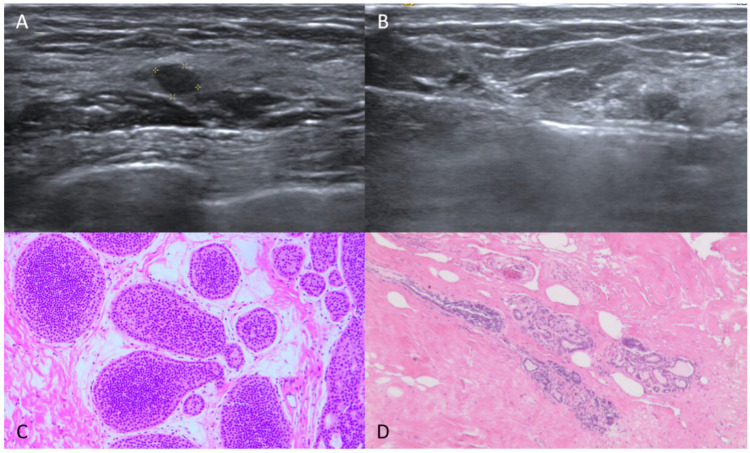
A 45-year-old lady with a hypo-echoic lesion in the left breast. (**A**) The tiny lesion (5 × 4 mm, asterisks mark the extent of the lesion) is oval, with a circumscribed margin. It was categorized as BI-RADS 3. (**B**) Ultrasound-guided vacuum-assisted excision was performed. (**C**) Pathology showed low grade ductal carcinoma in situ. (**D**) Post-surgery showed no residual carcinoma.

**Figure 3:**
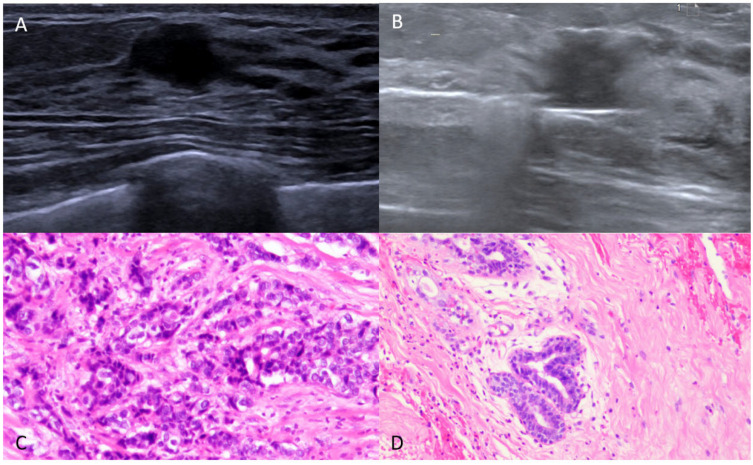
A 34-year-old lady with a hypo-echoic lesion in the left breast. (**A**) The lesion is irregular, with duct change. It was categorized as BI-RADS 4A. (**B**) Ultrasound-guided vacuum-assisted excision was performed. (**C**) Pathology showed invasive ductal carcinoma (grade III). (**D**) Post-surgery showed intermediate–high-grade ductal carcinoma in situ.

Our study retrospectively analyzed a group of patients with incidentally found breast cancer. The size, depth, and pathology of lesions derived by VAE were correlated with post-surgery residues. Lesions with a maximum diameter of less than 10 mm, a depth greater than 5 mm, and a pathology of ductal carcinoma in situ by VAE were more likely to be completely excised by vacuum-assisted excision without residue (Figure 2 and Figure 3).

The effectiveness of the EnCor System has been demonstrated in previous studies [13]. The large size of the needle (7G) used in the study facilitated rapid excision of the lesions, resulting in a sensitive predictive value for the presence of malignancy. Complete ultrasound-guided, vacuum-assisted excision is more accurate in histological diagnosis, minimizing the risk of pathological underestimation [1,14]. This was also confirmed in our study. No lesions with post-surgery residual cancer were found to be upgraded.

In our study, 72% of all incidentally found breast cancer was completely excised, and the residual rate was only 28%. The residual rate was much lower than that reported by X.F. He et al. [15]. In their study, the residual tumor rate after VAE was as high as 62.7%. Therefore, they concluded that minimally invasive surgery in breast cancer excision was invalid due to a high residual tumor rate. The lesion dimensions in the two studies were configured differently, and the tumor stages were entirely distinct. The lesions enrolled in our study were comparatively small and at a relatively early stage. Moreover, the data used in [15] were collected between 2010 and 2015. In the decade since, significant advancements have been made in both ultrasound diagnosis of breast lesions and VAE techniques, both of which might greatly elevate complete resection rates.

Incidentally found breast cancer is reported in clinic, but at a relatively low rate, owing to rapid progress in breast imaging. The addition of elastography and contrast-enhanced ultrasound has helped in selecting out malignancy from lesions categorized as BI-RADS 3 or BI-RADS 4A [16,17,18,19]. However, it is not routine to place a marking clip after each vacuum-assisted excision, and this leads to a social medical resource burden. The goal of breast-conserving surgery is to achieve negative margins with a satisfactory cosmetic result. In cases where a lesion is palpable or can be seen directly, it is easier for the surgeon to determine the surgery margin. In cases of incidental carcinomas which are supposed to be fully excised by vacuum-assisted excision, the question arises of how to arrange the margin. In such cases, hematoma targeting might be the preference. Hematoma was the complication most frequently found by ultrasound after vacuum-assisted excision. This facilitated location of the original incidentally found breast cancer. However, the healing rate was correlated with the location and size of the lesion, as well as compression tension post excision [20]. In our study, only 38.5% of the lesions were found with hematoma. In cases where hematoma was not seen on ultrasound, we found that several factors directly affected post-surgery residue, a finding which might help surgical planning.

In a study carried out by Pan et al., 61 malignant lesions were identified in a total of 5232 ultrasound-guided vacuum-assisted procedures, giving an incidence rate of about 1.2% [21]. Similarly, a 1.0% rate of malignancy was found in our research. All 53 instances of incidental cancer were at stage 0(TisN0M0) or stage 1A(T1N0M0). No positive lymph nodes were found during subsequent surgery. This inferred that all cases of incidentally found cancer were at an early stage, with low risk of metastasis, in line with the findings reported by Kong Y. et al. [11]. Our study also demonstrated that a higher complete resection rate of VAE was associated with DCIS lesions, consistent with the research findings of T. Perretta [22]. In Michaela Björnström’s study, although the lesions were small (≤10 mm), none could be completely resected by VAE because they were all invasive carcinomas. Mhairi Mactier et al. suggested that a minimally invasive technique like vacuum-assisted excision might offer a suitable alternative to surgical excision for low-risk, early breast cancers, particularly those involving small, good-prognosis tumors [8]. Many small tumors with favorable biologic features do not progress to large tumors within the lifetime of the patient [23]. Hence, these early-stage tumors might presumably be treated less invasively, without any need for surgery.

The study of Jiang Y. et al. showed that a larger lesion size may increase the likelihood of residual lesions remaining following ultrasound-guided vacuum-assisted excision. Their study concentrated on benign lesions and used follow-up as an index [24]. Likewise, in a study by Valadares et al., it was found that most cases without post-surgery residue involved tumors smaller than 10 mm [25]. In line with previous findings, our study also concluded that lesion dimension plays an important role in achieving complete excision [5,6,22]. Contrarily, however, Wang et al. stated that lesion size was not a key factor affecting complete excision. Their study considered lesions which were mostly benign, with only three being malignant [7]. Benign and malignant lesions have different essential biological characteristics. Benign lesions usually grow expansively while malignant lesions grow infiltratively. Thus, malignant lesions with larger size are more likely to found in residue following VAE.

Lesion depth contributed to cancer residue as well. Owing to the comparatively large suction power of the vacuum-assisted system, superficial lesions were handled with more care. Physical isolation by saline and light pressure were used for skin protection. Consequently, these might have also resulted in tumor residue.

Inevitably, the retrospective single-center nature of our study involved some limitations. First, the small sample size limited subgroup analysis of meaningful factors correlated with residual cancer. Additionally, it limited the generalization of the results. Second, the lesions were only evaluated by B-mode ultrasound and color Doppler ultrasound, and these could not provide additional information about lesion stiffness and microcirculation. Third, all patients were treated under ultrasound guidance. The stereotactic vacuum-assisted procedure was not taken into account.

## 5. Conclusions

In conclusion, our study summarizes those factors which might be correlated with post-surgery residue in cases of incidentally found breast cancer. These could be used by surgeons to schedule cost-effective intervention correctly and thereby elevate the success rate of one-time breast-conserving surgery with minimal resection. We also provide evidence that, in early-stage breast cancer, lesions could be potentially completely excised using the vacuum-assisted procedure.

## Figures and Tables

**Table 1:** Pathological characteristics of 53 cases of incidentally found breast cancer.

Pathology of Vacuum-Assisted Excision	Number	Post-Surgery Pathology	Number
Ductal carcinoma in situ	28	Residual cancer	15
Invasive ductal carcinoma	12	No residual cancer	38
Lobular carcinoma in situ	5		
Intraductal papillary carcinoma	4		
Invasive lobular carcinoma	2		
Mucinous carcinoma	2		

**Table 2:** Ultrasound-pathological characteristics correlated with post-surgery residue.

Characteristic	Residual Cancer	No Residual Cancer	*p*-Value
Patient age			
≤40	3 (20%)	11(28.9%)	0.749
>40	12(80%)	27(71.1%)	
Maximal diameter(mm)			
≤10	4(26.7%)	26(68.4%)	0.014
>10	11(73.4%)	12(31.6%)	
Location of the lesion			
Areola area	4(26.7%)	7(18.4%)	0.771
Non-areola area	11(73.3%)	31(81.6%)	
Depth of lesion(mm)			
≤5	10(66.7%)	11(28.9%)	0.027
>5	5(33.3%)	27(71.1%)	
Adjacent tissue			
Breast glands	4(26.7%)	21(55.3%)	0.116
Fat or muscle	11(73.3%)	17(44.7%)	
BI-RADS category			
BI-RADS 3	1(6.7%)	5(13.2%)	0.849
BI-RADS 4A	14(93.3%)	33(86.8%)	
Adler’s grade			
0	13(86.7%)	36(94.6%)	0.671
1	2(13.3%)	2(5.4%)	
Pathology of vacuum-assisted excision			
Carcinoma in situ	5(33.3%)	32(84.2%)	0.001
Non-carcinoma in situ	10(66.7%)	6(15.8%)	

Data are numbers of patients, with percentages in parentheses.

## Data Availability

Due to privacy requirements, the data presented in this study are available on request from the corresponding author.

## Abbreviations

The following abbreviations are used in this manuscript:

BI-RADS	Breast Imaging Reporting and Data System
VAE	Vacuum-Assisted Excision
IQRs	Interquartile Ranges
